# Differential Effects of Chronic Methamphetamine Treatment on High-Frequency Oscillations and Responses to Acute Methamphetamine and NMDA Receptor Blockade in Conscious Mice

**DOI:** 10.3390/brainsci12111503

**Published:** 2022-11-05

**Authors:** Matthew R. Hudson, Joshua Foreman, Gil Rind, Elizabeth E. Manning, Nigel C. Jones, Maarten van den Buuse

**Affiliations:** 1 Department of Medicine, Royal Melbourne Hospital, The University of Melbourne, Melbourne, VIC 3052, Australia; 2 Department of Neuroscience, Central Clinical School, Monash University, Melbourne, VIC 3004, Australia; 3 Florey Institute for Neuroscience and Mental Health, University of Melbourne, Melbourne, VIC 3052, Australia; 4 Centre for Eye Research Australia, Ophthalmology Eye and Ear Hospital, Melbourne, VIC 3002, Australia; 5 School of Biomedical Science and Pharmacy, The University of Newcastle, Callaghan, NSW 2308, Australia; 6 Department of Neurology, The Alfred Hospital, Commercial Road, Melbourne, VIC 3004, Australia; 7 School of Psychology and Public Health, La Trobe University, Melbourne, VIC 3086, Australia; 8 Department of Pharmacology, University of Melbourne, Melbourne, VIC 3052, Australia

**Keywords:** methamphetamine, mice, neural oscillations, gamma power, sensitisation

## Abstract

Dysregulation of high-frequency neuronal oscillations has been implicated in the pathophysiology of schizophrenia. Chronic methamphetamine (METH) use can induce psychosis similar to paranoid schizophrenia. The current study in mice aimed to determine the effect of chronic METH treatment on ongoing and evoked neuronal oscillations. C57BL/6 mice were treated with METH or vehicle control for three weeks and implanted with extradural recording electrodes. Two weeks after the last METH injection, mice underwent three EEG recording sessions to measure ongoing and auditory-evoked gamma and beta oscillatory power in response to an acute challenge with METH (2 mg/kg), the NMDA receptor antagonist MK-801 (0.3 mg/kg), or saline control. A separate group of mice pretreated with METH showed significantly greater locomotor hyperactivity to an acute METH challenge, confirming long-term sensitisation. Chronic METH did not affect ongoing or evoked gamma or beta power. Acute MK-801 challenge reduced ongoing beta power whereas acute METH challenge significantly increased ongoing gamma power. Both MK-801 and METH challenge suppressed evoked gamma power. Chronic METH treatment did not modulate these acute drug effects. There were minor effects of chronic METH and acute METH and MK-801 on selected components of event-related potential (ERP) waves. In conclusion, chronic METH treatment did not exert neuroplastic effects on the regulation of cortical gamma oscillations in a manner consistent with schizophrenia, despite causing behavioural sensitisation.

## 1. Introduction

Methamphetamine (METH) is a highly addictive amphetamine analogue, the chronic use of which is associated with several physiological, psychological and psychiatric effects [1,2]. The neurochemical mechanism of METH action includes an acute increase in dopamine release in the brain by targeting the plasmalemmal dopamine transporter and the vesicular monoamine transporter-2 [3,4]. Chronic METH use is associated with several adaptive changes in the brain, including functional sensitisation of its effects [5,6] despite concurrent decreases in tissue dopamine concentration and dopamine transporter density [7,8]. This sensitization is implicated in the development of METH psychosis, a severe side-effect of its long-term use which includes hallucinations, delusions and disorganized thought [9,10]. The symptom profile of METH psychosis overlaps with that of schizophrenia, although there are some distinct differences [10]. Similar sensitization of acute psychostimulant-induced dopamine release has been described in subjects with first-episode psychosis and those previously administered amphetamine repeatedly [11,12,13] and psychostimulant-induced psychosis is associated with a high risk of transition to schizophrenia [14,15]. These findings suggest that the effects of chronic METH can be used as a model to understand the underlying mechanisms involved in neurochemical, behavioural and neurophysiological aspects of schizophrenia [16]. Indeed we have previously used a chronic METH treatment protocol in mouse models of schizophrenia risk factors, such as reelin [17] and brain-derived neurotrophic factor (BDNF) [18,19,20,21]. 

Several studies have shown abnormalities in neural oscillations and synchrony in schizophrenia [22]. Such oscillations are an important mechanism to establish precise temporal relationships between neuronal responses and are implicated in memory, perception and consciousness [23,24]. High-frequency oscillations can be isolated into distinct frequency bands, including beta (20–30 Hz) and gamma (30–80 Hz); certain band-specific phenotypes, including increased ongoing, but reduced evoked gamma oscillations, have been associated with impaired function of parvalbumin (PV)-positive GABAergic interneurons in schizophrenia [25]. It is well-known that acute treatment with METH or amphetamine increases gamma oscillations in the nucleus accumbens [26], neocortex [27] and hippocampus [28], and chronic pretreatment with amphetamine may induce selected changes in EEG frequency bands in rats [29,30]. However, no previous studies have investigated the effect of chronic METH on ongoing and evoked oscillations in mice [31], the most widely used species for genetic modifications. In the present study, we therefore undertook a detailed assessment of the effect of chronic METH on different characteristics of ongoing and evoked oscillations in conscious mice, including components of event-related potentials (ERPs). Acute treatment with METH was used to verify any functional sensitisation of these measures. We also investigated the interaction with acute treatment with the N-methyl-D-aspartate (NMDA) receptor antagonist, MK-801, as it is widely considered that NMDA receptor hypofunction may play an important role in the pathophysiology of schizophrenia [32,33]. MK-801 and other NMDA receptor antagonists induce increases in ongoing gamma and beta oscillations and reduce evoked responses [34,35,36], similar to changes seen in schizophrenia [37]. The prediction was that chronic METH would induce schizophrenia-like effects on oscillations, specifically an increase of ongoing gamma power and a decrease of evoked gamma power. We furthermore predicted that acute administration of METH and MK-801 would enhance ongoing gamma power and suppress evoked gamma power, and that these effects would be enhanced in mice pretreated with METH.

## 2. Materials and Methods

### 2.1. Animals

**Figure 1:**
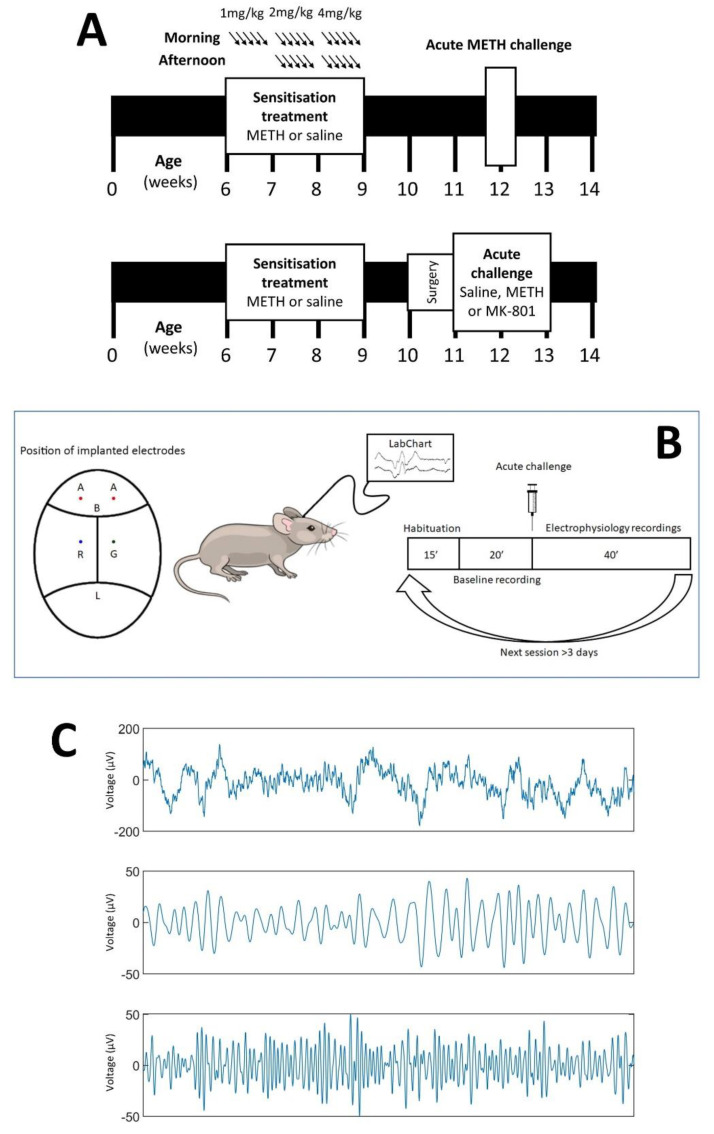
(**A**): Experimental timeline. Starting at 6 weeks of age, mice were treated with an escalating dosage regimen of METH or saline control each weekday for 3 weeks. The behavioural cohort (top) was subsequently tested at 12 weeks of age for the effect of an acute METH challenge on locomotor activity. The EEG cohort (bottom) underwent stereotactic surgery at 10 weeks of age to implant recording electrodes. (**B**): Location on the mouse skull where the electrodes are implanted (A = active; R = reference; G = ground), and a schematic of the experimental setup and daily procedure for recording. Over two weeks, three EEG sessions were conducted, during which the effect of METH or MK-801 was compared to saline control. (**C**): Example of a representative 2 s EEG recording at baseline. Top trace is unfiltered signal, middle trace is beta band (20–30 Hz) filtered and bottom trace is gamma band (30–80 Hz) filtered. Vertical scale depicts voltage (µV).

For the electrophysiological studies, we used a total of 18 male C57Bl/6 mice which were bred in the facility at the Melbourne Brain Centre, Parkville, Victoria, Australia. To confirm behavioural sensitisation following chronic METH treatment, we used a separate cohort of 26 male mice from the same breeding colony (Figure 1). Mice were group-housed in individually-ventilated cages (IVC, Tecniplast, Buguggiate, Italy) with ad libitum access to standard chow and water. Mice that underwent electrode implantation surgery were individually housed following surgery. All experiments were conducted in accordance with the Australian Code of Practice for the Care and Use of Animals for Scientific Purposes as set out by the NHMRC of Australia and approved by the Florey Institute of Neuroscience and Mental Health Animal Ethics Committee (#13–073-FNI).

### 2.2. Chronic METH Exposure Paradigm

All mice were randomly allocated to receive chronic treatment with either METH (National Measurement Institute, Canberra, Australia, *n* = 10) or saline vehicle (0.9% sodium chloride, *n* = 8), beginning at 6 weeks of age (Figure 1A), as previously described [19,20]. Briefly, during the first week, mice were given an intraperitoneal (IP) injection of 1 mg/kg of METH or equivalent dose of saline once in the morning every day for five days. In the second week of treatment, mice were given an IP injection of 2 mg/kg of METH or saline twice daily for five days, around 9 a.m. and around 5 p.m. In the final week, mice were given 4 mg/kg METH or saline twice daily for five days. All animals were then allowed at least one week to recover with access to food and water ad libitum.

### 2.3. Validation of Sensitisation

To confirm behavioural sensitisation following chronic METH treatment, mice from the behavioural cohort (Figure 1A, top) were placed individually into locomotor photocell chambers (Med Associates, St. Albans, VT, USA). Following one hour of habituation, the animals were then injected with 5 mg/kg METH and their movements were recorded for one hour. Ambulatory distance travelled over the post-injection hour was compared between mice chronically treated with saline and mice chronically treated with METH (*n* = 13 in each group). We have previously observed sensitisation in other mice cohorts using a 1, 3 and 5 mg/kg METH challenge [17,18,21,38,39,40]. Because of their electrode instrumentation, the mice from the electrophysiology cohort were not used for behavioural testing.

### 2.4. Electrode Implantation Surgery

Electrode implantation surgery was conducted to facilitate EEG recording [34,41]. Under isoflurane anaesthesia (Abbott Australasia, Doncaster, VIC, Australia), mice from the electrophysiology cohort (Figure 1A, bottom) were gently positioned in a stereotaxic frame and a midline incision was made in the skin to expose the skull. Burr holes were made in the skull using an electric drill, located at ±2 mm lateral and 2 mm anterior to bregma (active electrodes), and ±2 mm lateral and 2.5 mm posterior to bregma (ground and reference). Gold ‘male’ connector electrodes previously soldered onto nickel alloy jeweller’s screws were screwed into these holes, and dental acrylic (Vertex Dental, Soesterberg, The Netherlands) was applied on the skull to secure the electrodes. Animals were recovered for one week before further study.

### 2.5. Electrophysiology Studies

At 11 weeks old, mice underwent electrophysiological recordings as previously described [42,43] (see Figure 1B). This firstly involved connecting wires from the recording electrodes to a Bio Amp amplifier (ADInstruments, Bella Vista, NSW, Australia), before being connected to a PowerLab analogue-to-digital converter (ADInstruments). The EEG data were recorded using LabChart V4.5 software (ADInstruments). We also used selective 50 Hz noise eliminators (Humbugs; Digitimer, Letchworth Garden City, UK) to reduce electrical mains interference. 

Once connected (Figure 1B), mice were placed in a Plexiglass cylinder within an automated startle box (SR-LAB, San Diego Instruments, San Diego, CA, USA) for 15 min habituation, with a background noise level set at 70 dB [44]. Mice were then presented a series of auditory stimuli which were time stamped to the electrophysiological recordings. Broadband stimuli were 20 ms long at 85 dB, delivered every six seconds, which persisted for the duration of the session. 

We recorded EEG responses for 20 min for baseline measurement, and then mice were injected IP with either MK-801 (0.3 mg/kg, Sigma-Aldrich, NSW, Australia), METH (2 mg/kg) or vehicle (0.9% sterile saline). Following injection, a further 40 min of pulses were delivered, after which the mice were removed from the chamber and disconnected. All mice received all acute drug injections (saline, MK-801, METH) in a pseudo-random order, with at least 3 days between drug treatments [45].

### 2.6. Electrophysiology Analyses 

**Figure 2:**
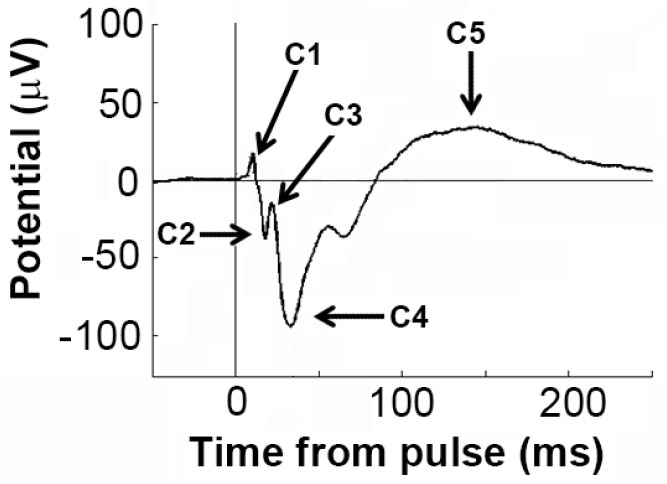
Representative ERP showing different wave components for analysis.

Analysis of EEG activity was done using MATLAB software (version 7.10.0, Natick, MA, USA: The MathWorks Inc., 2010; [46]). Prior to analysis, EEG was visually inspected to allow for rejection of noise or movement artefacts. From the clean EEG recordings, we performed two analyses which were compared between the groups, as described previously [44,47,48]: (1) spontaneous, or ongoing, oscillations in the beta (20–30 Hz) and gamma (30–80 Hz) high-frequency bands; (2) evoked responses triggered by the intermittent acoustic stimuli, incorporating assessment of changes in the power of beta and gamma oscillations triggered by the stimuli, as well as the individual components of the event-related potential (ERP, see Figure 2). We only calculated data acquired after injection, and data from left and right hemispheres were averaged. 

For assessment of electrophysiological responses elicited by the auditory stimuli, epochs (−500 ms to +500 ms relative to auditory stimulus) were first extracted and then averaged in the time domain for each session to compute the ERP. We consistently observed five components of the ERP observed in saline-treated mice (C1-C5), and we quantified the latency and amplitude of these peaks using custom-designed MATLAB scripts [44]. In some treated mice, there was no clearly evident peak at C2 and C3—in these cases, no data were entered.

Subsequently, using the same epochs evoked oscillatory power was computed using morlet wavelet decomposition. This analysis was achieved using the *newtimef* function from the EEGlab toolbox [49]. Briefly, the event-related spectral perturbation (ERSP) was computed at 180 linearly spaced frequencies from 1 to 200 Hz with three wavelet cycles for the lowest frequency (1 Hz), increasing to 10 cycles for highest frequency (200 Hz). Oscillatory power of the evoked responses was then calculated by averaging the ERSP in the different frequency bands of interest (β: 20–30 Hz; γ: 30–80 Hz). By subtracting baseline power (that occurring in the 300 ms immediately prior to the auditory pulse) from responses which occurred in the first 100 ms after the auditory stimulus (i.e., baseline-corrected), auditory evoked power was calculated.

For spontaneous oscillatory calculations, Fast-Fourier Transformation was performed on 3 sec epochs prior to each pulse using a 0.2441 Hz Hanning window. Data from these epochs were averaged to determine overall spectral power, which was broken down into individual frequency bands. EEGLab (v12; SCCN, University of California, San Diego, CA, USA) toolbox was used for these analyses. 

### 2.7. Statistical Analysis

Electrophysiological outcomes were compared using mixed-model ANOVA with repeated measures, with independent variables being sensitisation and acute drug treatment. Locomotor activity over one hour following injection of a challenge dose of METH was compared using Student’s t-test. In all cases, statistical significance was defined as *p* < 0.05. Analyses were performed using Graphpad Prism 8.0 (La Jolla, CA, USA), and all data represent mean ± SEM.

## 3. Results

### 3.1. METH Pretreatment Results in Sensitisation of Acute METH-Induced Locomotor Hyperactivity

**Figure 3:**
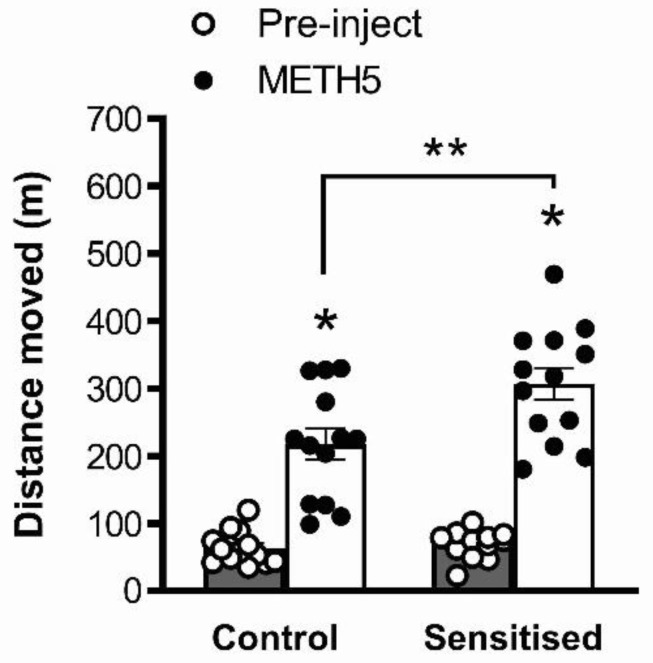
Acute METH-induced locomotor hyperactivity was significantly increased in METH-sensitised mice compared to vehicle-treated controls. * *p* < 0.05 for difference with pre-injection distance moved; ** *p* < 0.05 for difference with controls. METH5 = 5 mg/kg METH. *n* = 13 control mice; *n* = 13 sensitised mice.

An acute challenge with 5 mg/kg METH induced significant locomotor hyperactivity (one hour post-injection distance moved vs. pre-injection distance moved, F(1,48) = 131.2, *p* < 0.001). This hyperactivity was significantly greater in METH-pretreated mice than in saline-treated controls (main effect of pretreatment, F(1,48) = 7.82, *p* = 0.0074; interaction, F(1,48) = 5.98, *p* = 0.0182, Figure 3). Post hoc analysis showed that METH-pretreated mice showed significantly greater locomotor distance moved compared to saline-pretreated mice following a METH challenge (*p* < 0.05), but not before acute treatment, showing behavioural sensitisation to METH similar to our previous studies with this treatment protocol [17,18,19,38]. 

### 3.2. Ongoing Beta, but Not Gamma Power, Is Reduced by MK-801 and Acute METH

**Figure 4:**
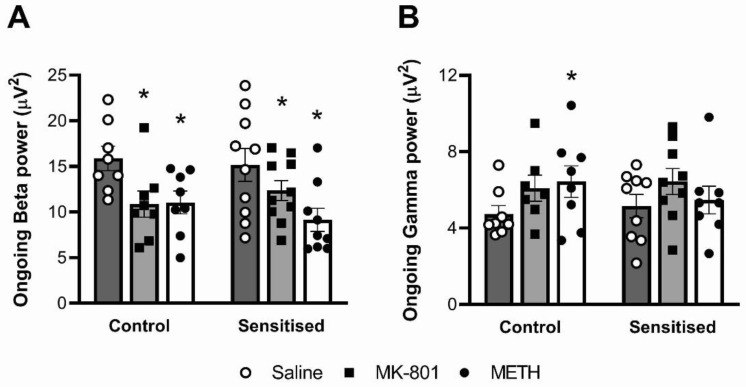
Effects of METH sensitisation and acute drug challenge on ongoing cortical oscillations. The animals were chronically treated with either saline vehicle (Control) or METH (Sensitised) and acutely treated with a saline, MK-801 and METH challenge. (**A**) Ongoing beta power was significantly reduced by acute MK-801 and METH treatment. (**B**) Ongoing gamma power was significantly increased by acute METH. There were no effects of METH pretreatment. * *p* < 0.05 for difference with acute saline injection. *n* = 8 control mice; *n* = 10 sensitised mice.

Acute drug challenge significantly reduced ongoing beta oscillatory power (F(2,31) = 23.70; *p* < 0.001), but METH sensitisation did not impact this outcome (Figure 4A). Post hoc comparison showed that in controls as well as sensitised mice, both acute MK-801 and METH challenges significantly reduced ongoing beta power compared to vehicle (*p* < 0.05). Ongoing gamma power was enhanced by acute drug challenge (F(2,27) = 3.77, *p* = 0.0358; no interactions). Post hoc comparison showed that, compared to acute saline, an acute METH challenge significantly increased ongoing gamma power in control mice (Figure 4B).

### 3.3. Auditory-Evoked Gamma Power, but Not Beta Power, Is Reduced by MK-801 and Acute METH

**Figure 5:**
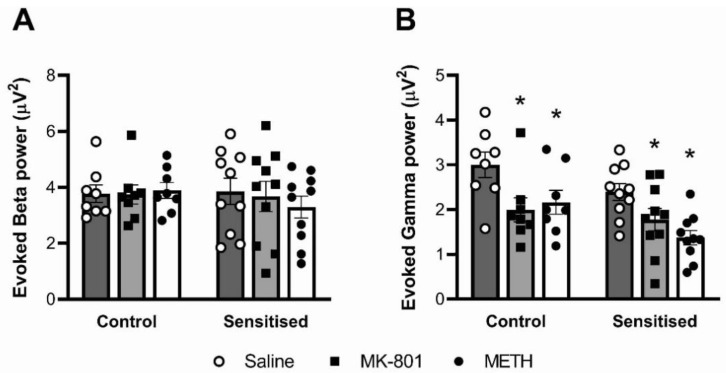
Effects of METH sensitisation and acute drug challenge on auditory-evoked cortical oscillations. Mice were chronically treated with either saline vehicle (Control) or METH (Sensitised) and acutely treated with a saline, MK-801 and METH challenge. (**A**) There were no effects of acute MK-801 or METH treatment on changes in beta power. (**B**) Both acute MK-801 and METH reduced evoked gamma power. There were no significant effects of METH pretreatment. * *p* < 0.05 for difference with acute saline injection. n = 8 control mice; n = 10 sensitised mice.

When assessing oscillatory power triggered by the auditory stimuli, we found that acute drug challenge significantly altered gamma power responses (F(2,32) = 21.79; *p* < 0.001) but had no effect on beta power responses (Figure 5). Post hoc analysis revealed that, in control as well as sensitised mice, both the METH and MK-801 challenge significantly reduced evoked gamma power compared to vehicle (*p* < 0.05). When comparing the effect of chronic METH, evoked gamma power was lower in sensitised mice than in controls, but this difference did not attain statistical significance (F(1,16) = 3.637; *p* = 0.075). 

### 3.4. Effects of Sensitisation to METH and Acute Drug Challenges on ERP Components

**Figure 6:**
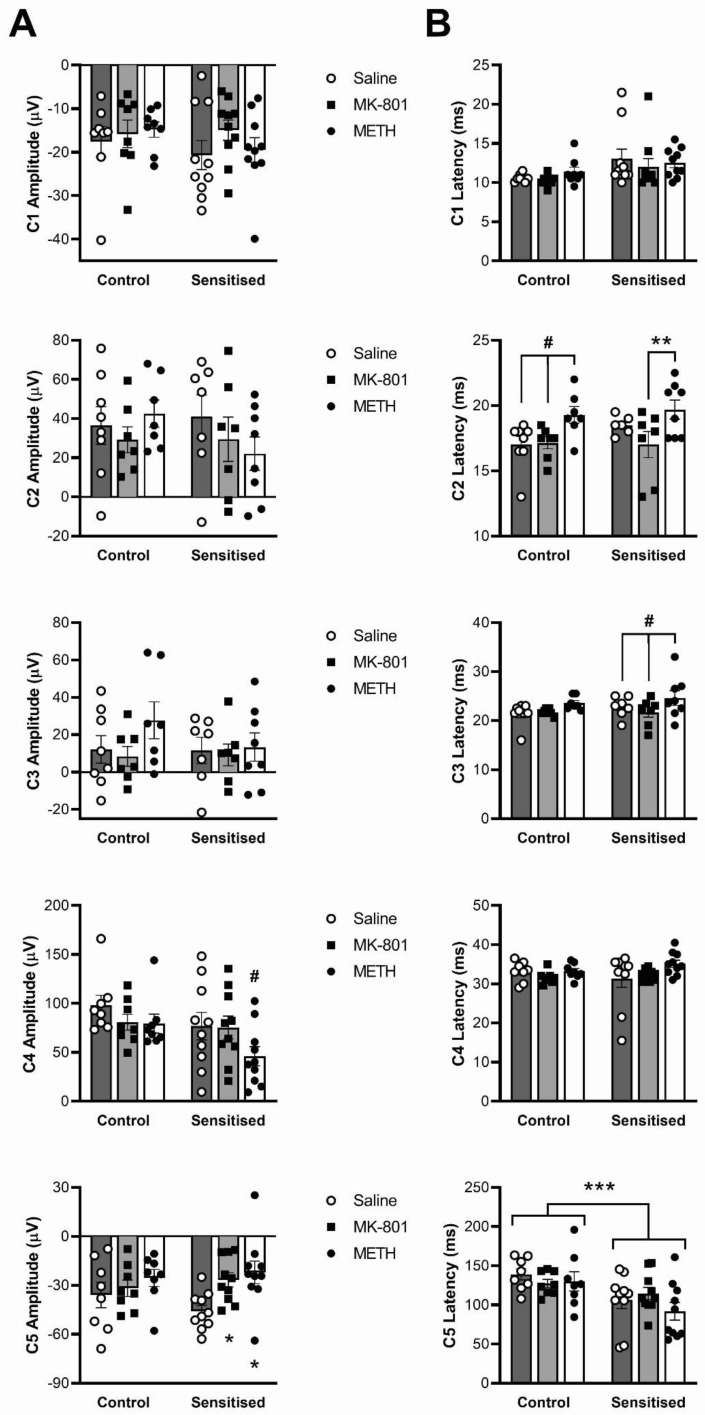
Amplitude (**A**) and latency (**B**) of ERP wave form components (see Figure 2) shown from top to bottom. The mice were chronically treated with either saline vehicle (Control) or METH (Sensitised) and acutely treated with a saline, MK-801 and METH challenge. * *p* < 0.05 for difference with acute saline treatment; ** *p* < 0.05 for difference with acute METH treatment; # *p* < 0.05 for difference with both acute saline and acute MK-801 treatment. *** *p* < 0.05 for differences between control and sensitised animals. *n* = 8 control mice; *n* = 10 sensitised mice.

We first observed and compared the ERP response between treatment groups. Table 1 provides the results of statistical analyses for the amplitudes and latencies of the five components of the ERP (see Figure 2). With regard to amplitude (Figure 6A), there was a significant effect of acute drug treatment on components C4 (F(2,32) = 7.51, *p* = 0.0021) and C5 (F(2,32) = 6.10, *p* = 0.0057) but there was no effect of chronic METH pretreatment. There was also no acute x chronic drug treatment effect interaction, suggesting that the effect of acute drug treatment was the same regardless of METH pretreatment. Nevertheless, post hoc analysis revealed that, for C4, amplitude was significantly reduced by METH compared to both saline and MK-801 treatment only in sensitised animals (*p* < 0.05). Similarly, post hoc analysis revealed that MK-801 and METH reduced the amplitude of the C5 peak only in sensitised animals (*p* < 0.05, Figure 6A).

With regard to the latencies of the ERP components (Figure 6B), there were significant effects of acute drug treatment for C2 (F(2,22) = 14.93; *p* < 0.001) and C3 (F(2,23) = 10.39; *p* = 0.006) but no acute x chronic drug treatment effect interactions. Post hoc analyses showed that, in controls, acute METH increased C2 latency compared to both saline and acute MK-801 treatment (*p* < 0.05) while in sensitised mice, MK-801 treatment induced significantly shorter C2 latency compared with acute METH (*p* < 0.05). Acute METH also significantly prolonged C3 latency compared with both saline and MK-801 only in sensitised animals (*p* < 0.05). 

In addition to acute drug effects, sensitisation by chronic METH pretreatment significantly shortened C5 latencies compared with control (main effect, F(1,16) = 7.18; *p* = 0.017) independent of acute treatment, and we observed a trend to prolonged C1 latency in sensitized mice (F(1,16) = 3.63; *p* = 0.075). None of the other comparisons or interactions attained statistical significance (Table 1).

## 4. Discussion

Chronic METH use can induce a psychosis similar to paranoid schizophrenia and both conditions are associated with dopaminergic sensitisation (see Introduction). Therefore, experimental studies on the long-term effects of METH may provide insight into pathophysiological mechanisms involved in the development of schizophrenia. In this study we focused on neural oscillations, alterations of which have been demonstrated in schizophrenia and implicated in the cognitive deficits found in this disorder, but have been little studied in METH sensitisation models in mice. We aimed to determine whether chronic METH treatment alters ongoing and evoked gamma oscillations in mice, and included the effects of acute METH-induced hyperdopaminergia and NMDA receptor hypofunction. The results show that chronic METH did not affect ongoing or evoked gamma power. Acute treatment with MK-801 and METH elevated ongoing gamma power and suppressed evoked gamma power but chronic METH treatment did not modulate these effects. Mice pretreated with METH showed the expected enhanced locomotor hyperactivity response to an acute METH challenge, confirming long-term sensitisation. These results suggest that chronic METH treatment does not affect the regulation of cortical gamma oscillations similar to schizophrenia.

Previous studies in schizophrenia animal models have shown significant changes in ongoing and evoked neuronal oscillations [34,36,44,47,50]. For example, mice heterozygous for the neuregulin 1 transmembrane domain showed elevated ongoing and reduced evoked gamma power [36]. Similarly, phospholipase C-beta1 knockout mice showed reduced evoked oscillatory power in both the beta and gamma frequency bands and increased ongoing beta power [47]. Few such studies have been done in METH sensitisation models. Janetsian et al. [29] used rats chronically treated with METH every other day for 13 days, followed by a one-week abstinence. Chronic METH did not alter the effect of acute METH to decrease gamma power [29], similar to the present study. However, these authors measured oscillations while the animals were under anaesthesia, conditions which can affect oscillatory activity and acute drug responses. In our study, mice were conscious. Lapish et al. [30] used conscious rats that had been treated with D-amphetamine every other day for nine days, followed by a 14-day non-treatment period. In addition to several changes in theta and delta frequencies (not studied here), amphetamine-treated rats showed a gradual increase in gamma and beta power in the prefrontal cortex and hippocampus [30]. An acute challenge with amphetamine did not alter gamma or beta power in either saline-pretreated or amphetamine-pretreated rats, suggesting lack of sensitisation to the drug [30]. Similar studies have not been done in mice, which is the focus of the present study. This also allowed us to compare our results with our previous studies in this species [36,44,47,48]. We included both ongoing and evoked oscillations and performed a detailed analysis of ERP components.

Decreased neuronal oscillations, particularly those in the gamma frequency band, have been associated with cognitive deficits in schizophrenia [25]. Indeed, we have shown that both cognitive deficits and changes in gamma power in a maternal immune activation mouse model of schizophrenia could be reversed by treatment with the selective estrogen receptor modulator (SERM), raloxifene [50]. Cognitive deficits have been described as well in individuals who use METH [1,51,52] and in animal models of METH abuse [53,54]. However, we recently performed a detailed analysis of a range of cognitive, affective and social behaviours in mice treated with the same METH protocol as in the present study [55]. Of particular relevance to the present results, METH-pretreated mice did not show changes in the Y-maze for short-term spatial memory, novel-object recognition test (NORT), context and cued fear conditioning, and prepulse inhibition (PPI) of acoustic startle. This lack of effect of chronic METH on cognitive measures would be consistent with the absence of changes in ongoing and evoked gamma power. It is possible that, in mice, higher or more prolonged METH treatment is required to induce parallel deficits in cognition and gamma power. Alternatively, unlike behavioural sensitisation, which persists for long periods of time, cognitive and oscillatory deficits may recover rapidly following chronic METH treatment. For example, a previous study found deficits in recognition memory at 1 day, but not 30 days after METH treatment [29].

Similar to gamma oscillations, changes in beta oscillations have been found in psychosis and schizophrenia [23,56,57] with increases in beta power correlating with positive symptoms [56,57]. While in the lack of changes in gamma power in the present study may be unsurprising given the absence of cognitive deficits in our METH model [55], the correlation of beta power and psychotic symptoms in patients with schizophrenia would predict similar effects of chronic METH. Nonetheless, in our mice, ongoing beta power was not altered following chronic METH pretreatment. Interestingly, acute challenge with MK-801 or METH, to model hypoactivation of NMDA receptors and hyperdopaminergia in psychosis, respectively [33], reduced beta power rather than increase it. This could indicate that the association of beta power and psychosis symptoms seen in patients with schizophrenia [56,57] does not apply to animal models that attempt to model this condition. However, in support of such a correlation, we previously showed increased beta power in two genetic animal models of schizophrenia, neuregulin-1 transmembrane domain heterozygous mice [36] and phospholipase C—beta1 knockout mice [47]. 

Detailed analysis of ERP wave components revealed several selective changes in both component peak amplitude and latency. ERP components are selectively altered in schizophrenia and have been suggested as a biomarker for the condition [16,58]. ERPs are generated by post-synaptic potentials resulting from neurotransmitter release in a large number of simultaneously firing cortical pyramidal cells. ERPs have been suggested to be a translational measure to assess sensory processing in a variety of species, even though specific ERP components may vary depending on species and even in different mouse strains [59]. The effects of acute MK-801 and METH on the amplitude of some and latency of other ERP components is in line with previous studies in mice and humans [60,61]. However, the only effect of chronic METH we observed was on the latency of one of the ERP components. Future studies should address the correlation of these individual ERP changes in response to acute and chronic treatments with specific behavioural deficits.

The study has a number of limitations. Firstly, we only used male mice. Gender differences have been described in the prevalence, symptom profile, and treatment response in schizophrenia [62]. Moreover, we recently found sex differences in the metabolism of METH [63], and female mice show greater locomotor hyperactivity to a given acute dose of METH than male mice [55]. Therefore, further studies on the long-term effects of chronic METH on neural oscillations should include female mice. Another limitation is that we did not measure gamma and beta power and ERPs during active behaviours. It is possible that ongoing and auditory-evoked changes in neural oscillations do not reveal functional differences between METH-pretreated mice and controls. For example, in a previous study in a maternal immune activation model of schizophrenia, we found deficiencies in local field potentials in the dorsal hippocampus specifically during decision making in a Y-maze memory task [50]. Future studies should therefore include electrophysiological recordings during selected behavioural tests.

In conclusion, we studied the effect of chronic METH treatment on ongoing and evoked neuronal oscillations, including the effects of pharmacologically induced hyperdopaminergia and NMDA receptor hypofunction, and ERP components. Chronic METH treatment did not exert neuroplastic effects on the regulation of cortical oscillations in a manner consistent with schizophrenia, despite causing behavioural sensitisation. 

## Figures and Tables

**Table 1:** Results of ANOVA for electrophysiological measures.

		Chronic METH		Acute drug		Interaction	
Variable		F	*p*	F	*p*	F	*p*
Ongoing power	Beta	0.050	0.826	23.70	**0.000**	2.255	0.122
	Gamma	0.192	0.667	2.819	0.077	2.417	0.108
Evoked power	Beta	0.153	0.701	0.307	0.687	0.760	0.476
	Gamma	3.637	0.075	21.79	**0.000**	1.805	0.181
Amplitude	C1	0.528	0.478	1.374	0.268	0.795	0.461
	C2	0.187	0.672	2.170	0.143	3.060	0.066
	C3	0.354	0.561	4.412	0.453	2.416	0.116
	C4	2.024	0.174	7.510	**0.002**	2.396	0.107
	C5	0.006	0.938	6.104	**0.006**	1.346	0.275
Latency	C1	3.634	0.075	1.009	0.376	0.617	0.546
	C2	0.507	0.487	14.93	**0.000**	0.802	0.461
	C3	0.291	0.597	10.39	**0.001**	0.515	0.604
	C4	0.060	0.810	2.064	0.144	1.313	0.283
	C5	7.718	**0.017**	1.335	0.278	1.523	0.233

Values in bold indicate significant *p*.

## Data Availability

Data are available upon request to the corresponding author.
